# Epstein–Barr Virus+ B Cells in Breast Cancer Immune Response: A Case Report

**DOI:** 10.3389/fimmu.2021.761798

**Published:** 2021-11-16

**Authors:** Andrea Aran, Vicente Peg, Rosa Maria Rabanal, Cristina Bernadó, Esther Zamora, Elisa Molina, Yago A. Arribas, Joaquín Arribas, José Pérez, Carme Roura-Mir, Montserrat Carrascal, Javier Cortés, Mercè Martí

**Affiliations:** ^1^ Immunology Unit, Department of Cell Biology, Physiology and Immunology, Institut de Biotecnologia i Biomedicina (IBB), Universitat Autònoma de Barcelona (UAB), Bellaterra, Spain; ^2^ Translational Molecular Pathology, Vall d’Hebron Institut de Recerca (VHIR), Barcelona, Spain; ^3^ Unitat de Patologia Murina i Comparada, Department of Animal Medicine and Surgery, Veterinary Faculty, Universitat Autònoma de Barcelona (UAB), Barcelona, Spain; ^4^ Preclinical and Translational Research Program, Vall d’Hebron Institute of Oncology (VHIO), Barcelona, Spain; ^5^ Breast Cancer Unit, Vall d’Hebron Institute of Oncology (VHIO), Hospital Universitari Vall d’Hebron, Barcelona, Spain; ^6^ Cancer Research Program, Hospital del Mar Medical Research Institute (IMIM), Barcelona, Spain; ^7^ Centro de Investigación Biomédica en Red de Cáncer, Madrid, Spain; ^8^ Institució Catalana de Recerca i Estudis Avançats (ICREA), Barcelona, Spain; ^9^ International Breast Cancer Center (BCC), Quironsalud Group, Barcelona, Spain; ^10^ Biological and Environmental Proteomics, Institute of Biomedical Research of Barcelona, Spanish National Research Council, Institut d'Investigacions Biomèdiques August Pi i Sunyer (IIBB-CSIC/IDIBAPS), Barcelona, Spain

**Keywords:** Epstein–Barr virus, B cells, T cells, breast cancer, TCR—T-cell receptor

## Abstract

EBV-specific T cells have been recently described to be involved in fatal encephalitis and myocarditis in cancer patients after immune checkpoint therapies. Here, we report the study of a human triple-negative breast cancer tumor (TNBC) and EBV-transformed B cells obtained from a patient-derived xenograft (PDX) that progressed into a lymphocytic neoplasm named xenograft-associated B-cell lymphoma (XABCL). T-cell receptor (TCR) high-throughput sequencing was performed to monitor the T-cell clonotypes present in the different samples. Forty-three T-cell clonotypes were found infiltrating the XABCL tissue after three passes in mice along 6 months. Eighteen of these (42%) were also found in the TNBC biopsy. TCR infiltrating the XABCL tissue showed a very restricted T-cell repertoire as compared with the biopsy-infiltrating T cells. Consequently, T cells derived from the TNBC biopsy were expanded in the presence of the B-cell line obtained from the XABCL (XABCL-LCL), after which the TCR repertoire obtained was again very restricted, i.e., only certain clonotypes were selected by the B cells. A number of these TCRs had previously been reported as sequences involved in infection, cancer, and/or autoimmunity. We then analyzed the immunopeptidome from the XABCL-LCL, to identify putative B-cell-associated peptides that might have been expanding these T cells. The HLA class I and class II-associated peptides from XABCL-LCL were then compared with published repertoires from LCL of different HLA typing. Proteins from the antigen processing and presentation pathway remained significantly enriched in the XABCL-LCL repertoire. Interestingly, some class II-presented peptides were derived from cancer-related proteins. These results suggest that bystander tumor-infiltrating EBV+ B cells acting as APC may be able to interact with tumor-infiltrating T cells and influence the TCR repertoire in the tumor site.

## 1 Introduction

The immune system plays a key role in cancer, but differences between individuals can lead to different outcomes. A high number of tumor-infiltrating lymphocytes (TIL) in triple-negative breast cancer (TNBC) are related to a better prognosis (1), although not in all patients (2). Several factors influence a good antitumoral immune response, such as the recognition of neoantigens, the presence of immunomodulatory mediators, or the cellular composition and diversity of TIL, including the presence of antigen-presenting cells (APC) such as dendritic cells (DC) or B cells. DC can migrate from the tumor site to the proximal lymph nodes to activate antigen-specific T cells, but the presence of APC in the tumor site is also necessary for the maintenance of cytotoxic T lymphocytes (CTL) and T helper (Th) cells. In the last years, tumor-associated B cells have been gaining interest and their presence has been associated with a good prognosis in breast cancer (3), colorectal cancer (4), ovarian cancer (5), hepatocellular carcinoma (6), soft sarcoma (7), and lung cancer (8), among others. Nevertheless, the role of B cells as active APC in the tumor site is not well established.

B cells are also the main targets of the Epstein–Barr virus (EBV) (9). Asymptomatic infections of young children by EBV are very common, so it is not surprising that EBV-infected cells (EBV+ B cells) are found among TIL (10) in many cancer types. EBV was the first recognized human oncovirus when it was isolated from a Burkitt lymphoma patient (9). Nowadays, the relationship between EBV infection and several lymphomas, i.e., Burkitt lymphoma (9, 11), Hodgkin’s lymphoma (12, 13), and posttransplant lymphoproliferative disorder (14, 15) is well established.

In the last years, the influence of endogenous virus in the outcome of carcinomas has been gaining evidence (16). Nevertheless, most of the studies are focused on the role of these viruses in the malignant transformation of epithelial cells, but the influence of virus-infected immune cells in the antitumoral response should also be considered. Even though epithelial cells can also be infected, B cells are the natural reservoir of EBV (17). Moreover, immunomodulatory effects are more likely to occur when the EBV infects B cells rather than epithelial cells since some tumor epithelial cells can downregulate the expression of HLA class I molecules (18).

EBV can be maintained for life in hosts because it has the capacity to persist in a latent state. It is well known that EBV can take advantage of the immunosuppression status of individuals (19) as suggested by the increase of lymphoma incidence in HIV-infected and organ-transplanted patients (20). This was supported by Mazar’s group who reported that 32% of patient-derived xenograft (PDX) tumors generated from epithelial tumor material can progress into EBV+ lymphocytic neoplasms (10)—or xenograft-associated B-cell lymphoma (XABCL) reported in other studies (21, 22)—evidencing the presence of EBV+ cells within the tumor material. The NOD SCID gamma (NSG) mice, used for PDX generation, are likely more susceptible to EBV-associated malignancies due to the lack of T-cell immune responses. Our proposal is that the presence of EBV+ B cells within a tumor may shape the antitumoral immune response.

EBV+ B cells in an infectious stage of the virus induce a strong T-cell-specific response (23). However, under the influence of the tumor microenvironment and/or of immunomodulatory treatments, the behavior, response, and repertoire of tumor-infiltrating cells—not only tumor-specific T cells but also bystander EBV-specific T cells—can be affected. This would give an opportunity for EBV in an infectious stage to survive but also could have long-term consequences affecting the immune system. The presence of EBV-specific T cells among tumor infiltrates has been described, and in two recent studies, their involvement in fatal inflammatory processes was suggested [myocarditis (24) and encephalitis (25)] in melanoma patients after immune checkpoint inhibitor (ICPI) therapies.

The aim of this study was to determine the relationship between tumor-infiltrating T and EBV+ B cells from a TNBC patient. To our knowledge, this is the first time that B cells generated in a XABCL have been used as an autologous EBV lymphoblastoid cell line (LCL) to study these B-cell–TIL interactions. T-cell receptor (TCR) and B-cell receptor (BCR) analyses were performed to study the presence of tumoral-infiltrating lymphocytes in the TNBC tumor as well as to monitor T-cell expansions. Our data as a first proof of concept have demonstrated that TIL derived from the breast cancer infiltrate can be maintained for at least 6 months in the XABCL, but also that certain clonotypes can be expanded *in vitro* in the presence of the established B-cell line derived from the XABCL (XABCL-LCL). Some of the TCRs involved have been previously described in cancer, but also in infections and autoimmunity. The whole peptide repertoire presented by HLA-ABC and HLA-DR from XABCL-LCL was also investigated and compared with LCL peptidomes from the literature. The characterization of the specific proteins from which these peptides were derived indicated that most of them were related with antigen processing and presentation pathways, suggesting that the XABCL-LCL can act as a strong APC. We have also assessed whether some of the proteins identified in the immunopeptidome had a relationship with cancer, and interestingly, most of the cancer-associated proteins were presented by class II molecules. In summary, this study suggests that EBV+ bystander B cells present in the tumor can interact with tumor-infiltrating T cells by acting as APC, thus participating in the antitumor response and influencing the outcome of the patient.

## 2 Material and Methods

### 2.1 Samples and Cell Cultures

#### 2.1.1 TNBC Biopsy

The biopsy Breast Cancer-Patient Sample-562 (BC-PS-562) was obtained from surplus hospital material, donated by the Vall d’Hebron Institute of Oncology (VHIO), by standard procedures with the appropriate approval of the Ethical and Scientific Committee of the institution. Consent was obtained from the patient according to local institutional review board requirement. The TNBC core biopsy was cut in several slices: five slices were frozen in liquid nitrogen and seven slices were cultured on a 48-well plate. Biopsy-derived T-cell clones were cultured in 1 ml of Iscove’s modified Dulbecco’s medium (IMDM) GlutaMAX™ (Gibco) supplemented with 1% penicillin–streptomycin, 10% of decomplemented human serum, and IL-2 (100 U/ml) (provided by the NIH). For this study, only the three tissue frozen slices were used as a source of the original tumoral tissue, and the cultures derived from four of the slices were used to study the T cells in culture derived from the explants.

#### 2.1.2 XABCL Generation

PDX mice (PDX-562) were generated by implanting a TNBC (BC-PS-562) core biopsy into NOD SCID mice. After three passes, the tumoral tissue was obtained. The tumor was collected in PBS and disaggregated by using a scalpel. Crushed tissue was resuspended in 5 ml of DMEM/F12 (Gibco) complemented with 10% of fetal bovine serum (Gibco), 1% L-glutamine (Biowest), 1% penicillin–streptomycin (Sigma-Aldrich), and 300 U/ml of collagenase type IA (Sigma-Aldrich) and incubated twice in a shaker 80×*g* and 37°C during 30 min. After centrifuging the disaggregated tissue, 100- and 40-μm pore cell strainers were used to obtain a uniform single-cell suspension from the cell pellet.

#### 2.1.3 XABCL-LCL Culture

After tissue digestion of the XABCL tumoral tissue, B cells were cultured and grown to a final concentration of 3 × 10^5^ cells/ml in T75 flasks using RPMI GlutaMAX™ (Gibco) with 1% penicillin and streptomycin (Sigma-Aldrich) and 10% of Fetal Bovine Serum (Gibco).

#### 2.1.4 TNBC-T Cells and XABCL-LCL Co-Cultures

To select XABCL-reactive T cells, TNBC-T cells from one of the tissue slices were grown for 10 days in culture medium without cell-specific stimulus. Cells were grown at a final concentration of 3 × 10^5^ cells/ml with IMDM GlutaMAX™ (Gibco) complemented with 10% of human serum, 1% penicillin–streptomycin (Sigma-Aldrich), and IL-2 at 100 U/ml in contact with XABCL-LCL B cells and allogeneic PBMCs, previously irradiated at 60 and 30 Gy, respectively. On the 7–10th day, cells were restimulated using anti-human CD3 and anti-CD28-coated Dynabeads (Gibco). As a control, cells were grown using a rapid expansion method (REM) (26). T cells (1–1.5 × 10^5^) were cultured in the presence of feeders (25 × 10^6^ 30 Gy irradiated PBMCs) in a T25 flask with 25 ml of complete medium (IMDM GlutaMAX™ with 1% P/S and 10% of decomplemented human serum) supplemented with 50 ng/ml of soluble anti-CD3 (OKT3) and IL-2 at 200 U/ml. On the fifth day, half of the medium was removed and replaced with fresh medium with IL-2 at 200 U/ml, and cells were collected 10–12 days after stimulation.

### 2.2 Tumoral and T- and B-Cell Presence in the Biopsy

#### 2.2.1 Immunohistochemistry

The presence of tumoral cells and infiltrating T and B cells in the biopsies was determined by hematoxylin and eosin (HE) and immunohistochemistry (IHC). Antibodies used for the paraffin-embedded sections of the TNBC biopsy were anti-human CD3 (clone 2GV6), anti-human CD4 (clone SP35), anti-human CD8 (clone SP57), and anti-human CD20 (clone L26) (Ventana Medical System, Inc.). IHC of the XABCL tissue was performed in samples fixed in 10% neutral buffered formalin. Transverse sections were used for processing. Morphological evaluation was performed on 5-µm paraffin-embedded sections, stained with HE. The primary antibodies used were rabbit polyclonal anti-human CD3 (Dako) and rabbit polyclonal anti-human CD20 (Thermo Fisher). Sections were incubated with a labeled polymer [anti-rabbit for CD3 and CD20 (Agilent-Dako)] according to the instructions of the manufacturer. IHC was completed using 3,3′-diaminobenzidine (DAB) and counterstaining in hematoxylin. Positive controls of the XABCL IHC were performed using normal mouse spleen and lymph node. In all the experiments, for the negative control, an isotype-specific immunoglobulin was used to substitute the primary antibody as negative control; no immunostaining was detected in these sections.

### 2.3 Characterization of XABCL-LCL and TNBC-T Cells

#### 2.3.1 Phenotypic Characterization of T and B Cells

Between 2 and 5 × 10^5^ of cells were stained with anti-human antibodies for 20 min at 4°C in the dark with PBS containing 2% FBS. After incubation, cells were washed twice, collected in 200 μl of PBS, and analyzed by flow cytometry.

For the XABCL-LCL phenotype analysis, anti-human antibodies used were PE-conjugated anti-CD19 (BD Pharmingen), PE-conjugated anti-CD20, FITC-conjugated anti-CD21, PE-Cy7-conjugated anti-CD38, APC-conjugated anti-HLA-ABC (BD Pharmingen), PE-conjugated anti-HLA-DR (BD Pharmingen), FITC-conjugated anti-CD80 (ImmunoTools), FITC-conjugated anti-CD86 (ImmunoTools), PE-conjugated anti-PD-1 (eBioscience), and FITC-conjugated anti-PD-L1 (BD Pharmingen). For the T-cell phenotype analysis, the following human antibodies were used: PerCP-conjugated anti-human CD3, PE-conjugated anti-human or FITC-conjugated anti-human CD4, and FITC-conjugated anti-human CD8 or APC-conjugated anti-human CD8 (BD Pharmingen). The flow cytometer used was BD FACS Canto. Analyses were performed by using the FlowJo and FACS Diva software.

#### 2.3.2 BCR and TCR High-Throughput Sequencing of TNBC, XABCL, TNBC-T Cells, and XABCL-LCL

DNA extraction from the TNBC and XABCL tissues and XABCL-LCL was carried out using a phenol–chloroform protocol. Tissue samples were disrupted by sonication. Amplification of BCR and TCR transcripts from DNA was performed using survey level ImmunoSEQ technology by Adaptive Biotechnologies. For the high-throughput sequencing (HTS) of TNBC-T cells, cells were counted and between 1 × 10^5^ and 1 × 10^6^ of T cells from the initial cultures and expanded T cells were used for RNA extraction. RNA was isolated using RNeasy Micro Kit and RNeasy Mini Kit (Qiagen), respectively, following the instructions of the manufacturer. An optional on-column DNase digestion was also performed during the RNA isolation using the RNase-Free DNase Set (Qiagen). RNA amounts and integrity were measured with Agilent 2100 bioanalyzer (Agilent Technologies). Human TCR profiling from TIL was assessed by using the SMARTer Human TCR a/b Profiling Kit (Takara Bio), which allows the complete capture of V(D)J variable regions of TCR transcripts and library production, directly indexed for sequencing. Both TCR-alpha and TCR-beta chain diversities were studied. Purification of amplified libraries was performed using Agencourt AMPure XP Beads (Beckman Coulter), following the commercial recommendations. Analysis and validations of libraries were carried out on an Agilent 2100 Bioanalyzer, using the DNA 1000 Kit (Agilent Technologies). Sequence was performed on an Illumina MiSeq sequencer using the 600-cycle MiSeq Reagent Kit v3 (Illumina) with paired-end 2 × 300 base pair reads.

#### 2.3.3 TCR and BCR Repertoire Analysis

TCR and BCR sequencing results obtained from the ImmunoSEQ analysis were evaluated by the ImmunoSEQ analyzer, v.3.0. TCR sequencing data obtained from the SMARTer Human TCR a/b Profiling Kit were evaluated by the MiXCR Immune Repertoire Analyzer (27). Raw data obtained from both technologies were processed using VDJTools (28). Non-productive clonotypes were excluded. The remaining repertoire data were collapsed by CDR3 amino acid sequences, i.e., clonotypes with different nucleotide sequences encoding the same CDR3 amino acid sequence were summed, and frequencies were recalculated. The different TCR and BCR sequencing results from each sample were compared to find out the common sequences shared by the XABCL, TNBC, and TNBC-T cells. Diversity and heat plot analyses were also performed using VDJTools (28). The TCR sequences obtained from this analysis were compared with the McPAS-TCR (29) database with a Levenshtein distance of 1. Figures were performed using the Graphpad Prism 7.0 software. UpSet plots were generated using R (version 3.6.1) (30) using the “dplyr,” (31) “RColorBrewer,” (32) and “UpSetR” (33) packages.

### 2.4 Characterization of the HLA-Associated Peptide Repertoire From the XABCL-LCL

#### 2.4.1 Purification of Peptide–HLA Complexes

Two 40 × 10^6^ pellets of XABCL-LCL were used to elute peptides presented by HLA-ABC and HLA-DR. The cellular lysis was performed as described by Heyder et al. (34) Briefly, the cell pellet was resuspended in lysis buffer [50 mM Tris–HCl (pH 8.0), 150 mM NaCl, 1× Complete protease inhibitor (Roche), and 5 mM EDTA]. Nuclei and cell debris were cleared by 2× 12 min centrifugations at 5,500×*g* after sonication (40% of amplitude of max. 130 W) and 10 min incubation at 4°C. Membranes collected from the supernatant were pelleted with 1 h ultracentrifugation at 72,000×*g* and 4°C and resuspended in a solubilization buffer [1% n-dodecyl-β-D-maltoside, 50 mM Tris–HCl (pH 8.0), 150 mM NaCl, 1× Complete protease inhibitor (Roche), and 5 mM EDTA]. Solubilized pelleted membranes were sonicated 4× 5 s (20% amplitude of max. 130 W) and incubated overnight at 4°C. Non-solubilized membranes were pelleted by ultracentrifugation at 55,000×*g* for 1 h at 4°C, and HLA complexes in the supernatant were then purified from the soluble fraction based on the Spetniak et al. protocol (35). Solubilized membranes were incubated overnight with 100 μl CNBr-activated sepharose beads (GE Healthcare Life Sciences) coupled to anti-HLA-ABC w6/32 or to the anti-HLA-DR B8.11.2 antibodies. Antibody-conjugated sepharose beads were washed 3× with 50 mM Tris–HCl (pH 8.0), 150 mM NaCl, and 0.5% n-dodecyl-β-D-maltoside and 3× with 50 mM Tris–HCl (pH 8.0) and 150 mM NaCl. Unspecific interactions were diminished by washing with a high-salt concentration buffer [50 mM Tris–HCl (pH 8.0), 0.5 M NaCl]. Peptide–HLA–DR complexes were eluted with 0.25% TFA after washing 3× with 50 mM Tris–HCl (pH 8.0) and 150 mM NaCl and 1× with 20 mM Tris–HCl (pH 8.0). The eluted complexes were cleared by SCX and a C18 tip before mass spectrometry.

#### 2.4.2 Peptide Identification by Mass Spectrometry

Samples were analyzed by liquid chromatography coupled to high-resolution mass spectrometry (LC-HRMS). The LTQ Orbitrap XL mass spectrometer (Thermo Fisher) equipped with a nanoESI source and coupled to an Agilent 1100 nanochromatographic system was used. LC separation was performed using a 140-min acetonitrile gradient. Analyses were performed in data-dependent mode at a target mass resolution of 60,000 (at *m*/*z* 400). Up to 10 of the most intense peaks with charge ≥2 and intensity above the 500-unit threshold were selected and fragmented by CID. To minimize the redundant selection of precursor ions, dynamic exclusion was set to 1 for 45 s. Raw data were processed using Proteome Discoverer 1.4 against the human and viral proteome database (UniProt/SwissProt) with the following parameters: no enzyme, 20 ppm precursor mass tolerance, 0.02 Da fragment tolerance, and variable modification of oxidized methionine (+16 Da). Peptide spectral matches were filtered at 1% FDR using Percolator.

#### 2.4.3 HLA-Derived Peptide Repertoire Analysis

Peptides identified were prefiltered by following these rules: elimination of I) peptides outside the range 8–13mer and peptides shorter than 10-mer peptides, for class I and class II analyses, respectively; II) keratin-derived peptides; and III) duplicate peptides with modifications. The subcellular location of the source proteins was extracted from the UniProt (36) database. Proteins that were found in more than one subcellular location were counted in both localizations. DAVID (37, 38), which executes a functional annotation clustering using different databases, was used for the KEGG Pathway Database pathway enrichment analysis. For the Gibbs clustering, peptides found in nested sets were excluded, leaving only the shortest peptide.

#### 2.4.4 Identification of Proteins in the XABCL-LCL HLA Peptide Repertoire

To exclude peptides from proteins commonly presented by LCL, the source proteins of the peptides obtained in the HLA-ABC analysis were compared with 26,305 source proteins, from 6 different studies and 7, 11, and 9 different HLA-A, B, and C alleles, respectively (39–44). For the HLA-DR peptides, 517 source proteins from 10 different studies and 11 different HLA-DR alleles found in the literature (45–54) were used. The assignment of the peptides to the corresponding allele was performed according to the following criterion. First, only peptides clustered by Gibbs Cluster 2.0 (55) were used. Then, peptides predicted as strong binders by the NetMHCIPan 4.1 Server and the NetMHCIIpan 4.0 Server (56) were considered. Peptides with a peptide spectral match (PSM) of 1 were excluded. The source proteins from the peptides obtained were examined in the Human Protein Atlas (57–59) to check for specificities in cancer. The number of proteins, peptides, and PSM was considered. All data were represented using the GraphPad Prism 7.0 software.

## 3 Results

### 3.1 The Tumor Generated in a PDX Derived From the Tumor of a TNBC Patient Was the Result of a Monoclonal Expansion of EBV+ Tumor-Infiltrating B Cells

A 44-year-old patient was diagnosed with TNBC. The presence of an infiltrating ductal carcinoma [histological grade 3, no vascular invasion, estrogen receptor (ER)-negative, progesterone receptor (PR)-negative, human epidermal growth factor 2 (HER2)-negative, and Ki67 70%] was confirmed by the Pathological Anatomy Unit of the Vall d’Hebron Institute of Oncology (Barcelona, Spain).

**Figure 1:**
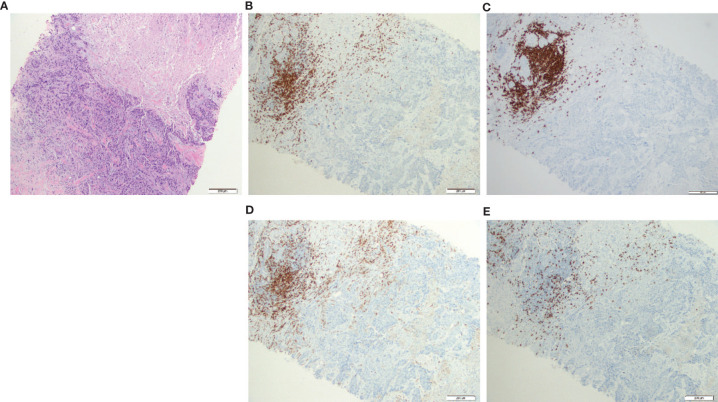
CD3+ T cells (both CD4+ and CD8+) and CD20+ B cells were present and colocalized in the patient biopsy. Hematoxylin and eosin (HE) staining and immunohistochemistry (IHC) from the triple-negative breast cancer (TNBC) core biopsy realized after diagnosis. **(A)** Histological section stain from breast carcinoma (HE ×200); **(B)** CD3 IHC stain in breast carcinoma highlighted numerous CD3+ T cells in the infiltrating front of neoplasia (×200); **(C)** CD20 IHC stain showed a similar distribution of CD20+ B cells compared with CD3+ T lymphocytes (×200); **(D)** CD4 IHC stain revealed that most lymphocytes are CD4+ T cells (×200); **(E)** CD8 IHC stain highlighted less CD8+ T cells but nearest to the tumor in the infiltrating front (×200).

Two core biopsies were obtained at the same time, i.e., 2 weeks after diagnosis and before neoadjuvant chemotherapy; one was inoculated in an NSG mouse to obtain a PDX and the other one was used to study the T-cell infiltration directly from the tissue or from the T-cell cultures (TNBC samples, from now on TNBC and TNBC-T cells, respectively). The TNBC (**Figure 1A**) was infiltrated with CD20+ B cells and CD3+ T cells (**Figures 1B, C**). A similar distribution was observed for both B and CD4+ T cells, suggesting that these cells could be interacting in the tumor site. Although there was a higher presence of CD4+ T cells (**Figure 1D**), CD8+ T cells were also found (**Figure 1E**).

**Figure 2:**
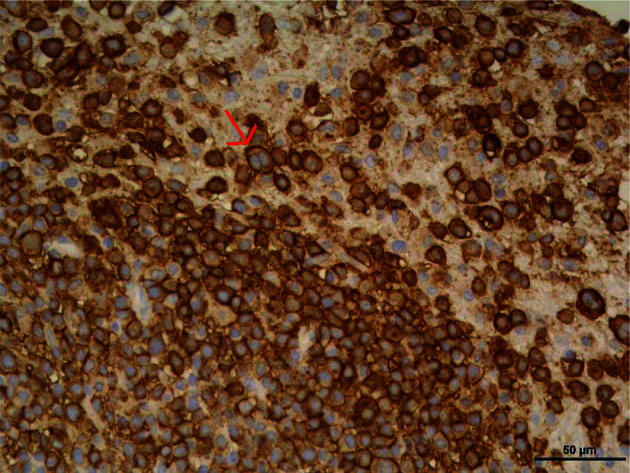
The tumor derived from a TNBC patient implanted in a PDX was a B-cell lymphocytic tumor (XABCL). CD20 immunohistochemical stain in the histological section revealed that most of the cells were CD20+ B cells. Cells with a bilobed nucleus, reminding of the Reed–Sternberg cells associated with Hodgkin’s lymphoma, were observed (pointed with a red arrow).

The tumor grown in the PDX mouse was obtained after three passes in mice. Cells from this culture did not show epithelial tumor cell line characteristics, but an LCL-like phenotype. A stable long-term cell line was established from the digested tumoral tissue (XABCL-LCL, from now on). The lymphocytic origin was confirmed by IHC (**Figure 2**), and not only most of the cells were CD20+, but also some of these cells presented a bilobed nucleus, reminding of the Reed–Sternberg cells associated with Hodgkin’s lymphoma (12). An HLA genotyping confirmed the patient cells as the origin of the XABCL tumor (HLA-A*02:01, A*30:02, B*18:01, B*49:01, Cw*05:01, Cw*12:03, DRB1*03:01, DRB1*11:01, DQB1*02:01, DQB1*03:01), and we confirmed that both the XABCL-LCL and the PBMCs from the patient—but not the TNBC-T cells—were EBV+ by qPCR (data not shown). The phenotypic analysis of the XABCL-LCL revealed a molecular pattern that resembled an activated germinal center B cell, i.e., CD19+, CD20+, CD21−, and CD38+, and they also expressed HLA-ABC and HLA-DR molecules as well as CD80, CD86, and PD-L1 (**Supplementary Figure 1**), confirming that these cells have antigen-presenting capacities, as it was expected in a B-cell line. We could not detect antibodies in the supernatant from the XABCL-LCL culture (data not shown).

A BCR HTS revealed that one single BCR sequence, i.e., CAKDVTDVSAYYYQAEYFPHW (IGHV03-23), represented more than the 97% of the repertoire in both samples, i.e., the XABCL tissue and the XABCL-LCL (**Supplementary Table 1**), confirming a monoclonal expansion produced before the *in vitro* cell line establishment. We performed the BCR HTS on the TNBC tissue and the same BCR sequence was found, confirming that the B cells that composed the XABCL were originally present within the tumor infiltrate of the patient and transformed into a monoclonal lymphocytic tumor in the mouse.

### 3.2 XABCL B Cells Can Maintain Tumor-Infiltrating T Cells *In Vivo* and *In Vitro*

Tissue sections were obtained from each pass in mice and the IHC of the XABCL tissue revealed some CD3+ T cells infiltrating the tumor after the first pass (**Supplementary Figure 2**). A TCR CDR3 HTS was performed to study which infiltrating T-cell clonotypes could have remained in the XABCL tissue, obtained from mice after the third pass. The analysis revealed 43 different T-cell clonotypes where the most prevalent clone represented 31.8% of the sample (**Supplementary Table 2**).

**Figure 3:**
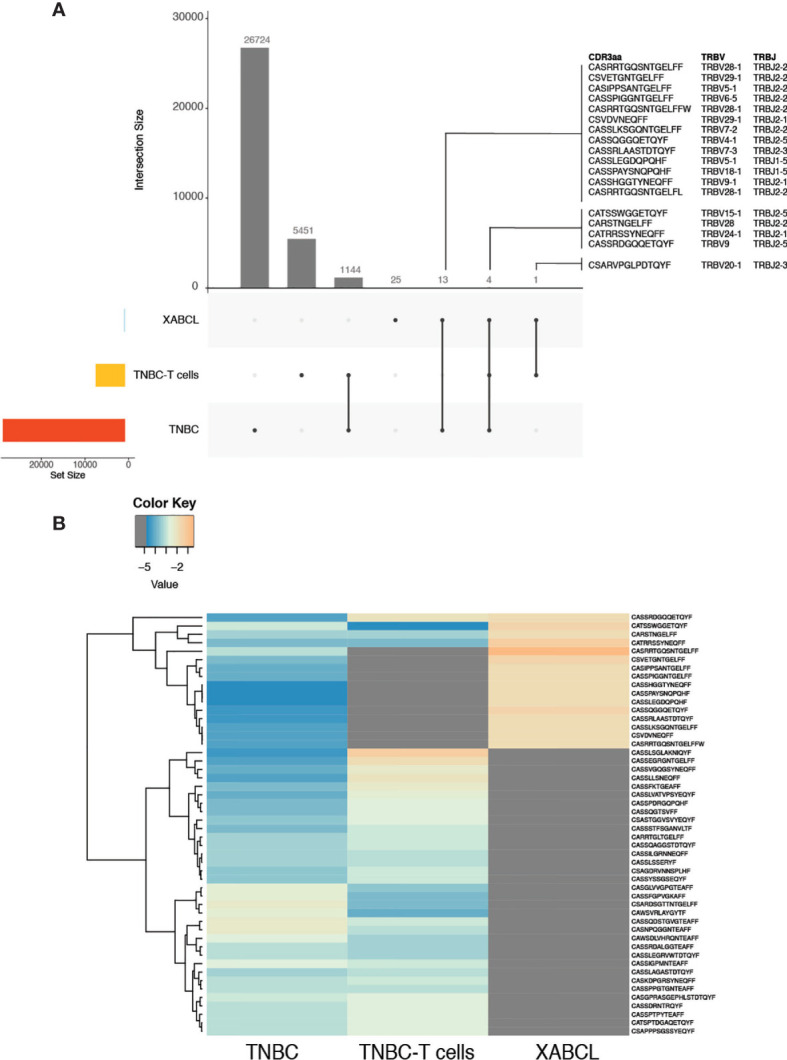
Some T-cell receptor (TCR) sequences from the XABCL tissue were present in the TNBC tissue and the TNBC-T cells, but the three samples presented different TCR repertoire patterns. **(A)** UpSet plots represent the number of shared TCR sequences between samples. Eighteen TCR sequences were found in the XABCL and the TNBC biopsy and/or the TNBC-T cells. Twenty-five sequences were exclusively found in the XABCL. **(B)** The heat plot of the 50 most abundant clonotypes in the shared samples revealed a global different pattern of the TCR repertoire infiltrating the XABCL.

To prove that the clonotypes found in the XABCL tissue came from the human biopsy, a TCR CDR3 HTS was also realized on the TNBC samples. As expected, many more clonotypes were identified: 26,724 from the TNBC and 5,451 in the TNBC-T cells. A total of 1,144 clonotypes were shared between these two sets (**Figure 3A** and **Supplementary Table 2**). Eighteen TCR sequences appeared in common between the TNBC samples and the XABCL tissue: 4 were shared by three samples, 13 were shared by TNBC and XABCL tissues, and only 1 sequence was exclusively found in the TNBC-T cells and the XABCL tissue. Only one of the four TNBC-T cell samples (slices in culture) analyzed (see *Material and Methods*) shared a TCR sequence with the XABCL.

A tracking heat plot of the 50 most abundant clonotypes—using samples with shared sequences—revealed a different pattern of TCR in the three sets, especially in the XABCL tissue (**Figure 3B**), i.e., the most abundant clonotypes infiltrating the XABCL after 6 months were not the most abundant in the TNBC tissue or in the TNBC-T cell sample. Thus, the XABCL model demonstrated that B cells can maintain some tumor-infiltrating T cells, confirming a possible role of B cells as APC in the tumor microenvironment.

**Figure 4:**
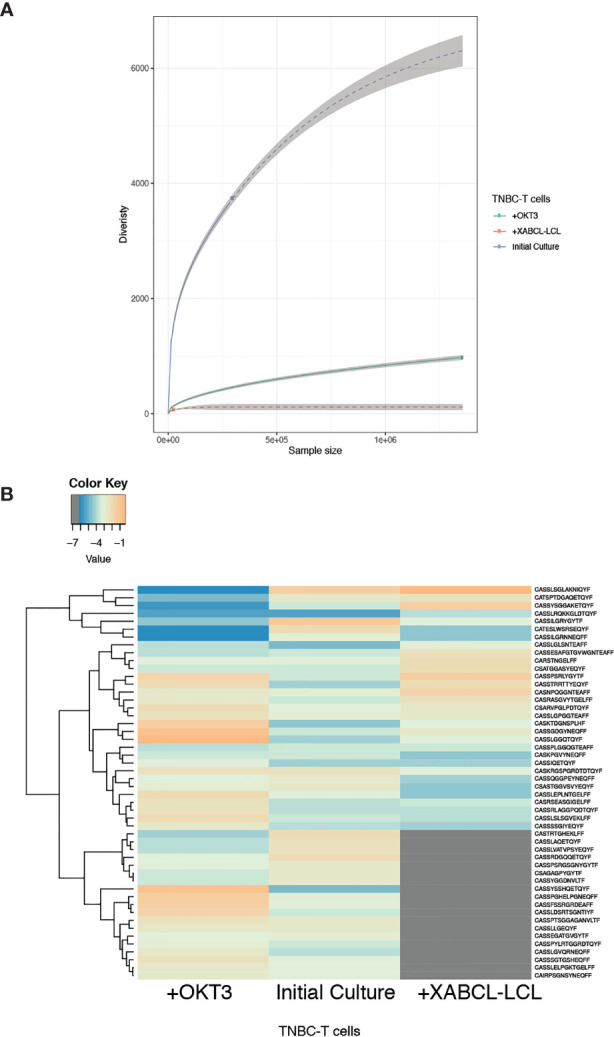
TNBC-T cells expanded *in vitro* in the presence of XABCL-LCL presented a highly restricted TCR repertoire with a different pattern. **(A)** The TCR repertoire size and diversity hugely decreased in the TNBC-T cell sample expanded in the presence of irradiated XABCL-LCL (60 Gy), compared with TNBC-T cells expanded with OKT3 (anti-human CD3). **(B)** The heat plot using the 50 most abundant clonotypes revealed that the TCR repertoire pattern of expanded TBNC-T cells was also different in the T cells grown with XABCL-LCL, instead of with OKT3, as several sequences were not found in common and different frequencies of the shared clonotypes were observed (**Supplementary Table 3**).

*In vitro* stimulation was performed by co-culturing TNBC-T cell with irradiated XABCL-LCL and comparing with control TNBC-T cells expanded in the presence of OKT3 (anti-human CD3). The TCR repertoire and T-cell phenotypic changes after co-culture were analyzed by TCR HTS and flow cytometry, respectively. A low number of sequences (73 clonotypes) with very low TCR diversity were obtained after co-culture (**Figure 4A** and **Supplementary Table 3**), also displaying a pattern different from the OKT3 control (**Figure 4B** and **Supplementary Figure 3A**).

Only two of the five shared sequences between XABCL tissue and TNBC-T cells (**Figure 3A**) were maintained, i.e., CARSTNGELFF (TRBV28) and CSARVPGLPDTQYF (TRBV20-1) (**Supplementary Figure 3B**). These two clonotypes were also found in the control T-cell cultures expanded with anti-CD3, but the CARSTNGELFF sequence represented a larger normalized fraction after the co-culture with the XABCL-LCL. Phenotypic analysis (**Supplementary Figure 3C**) showed little differences other than a decrease of the double-negative (DN) T-cell population, which represented 41.8% of T cells in the initial culture and only 1.34% after co-culture. This subset appeared to be replaced by CD4+ T cells, varying from 15% in the initial culture to 43.6% after co-culture. CD8+ T cells displayed similar frequencies in both samples: 40.6% and 52.1% in the initial culture and after co-culture, respectively. Thus, the results of the *in vitro* experiments confirmed that, even after transformed to an LCL, B cells derived from the XABCL tumor influenced the TCR repertoire of the infiltrating T cells.

### 3.3 Previously Reported TCR Sequences Are Maintained by XABCL B Cells

**Figure 5:**
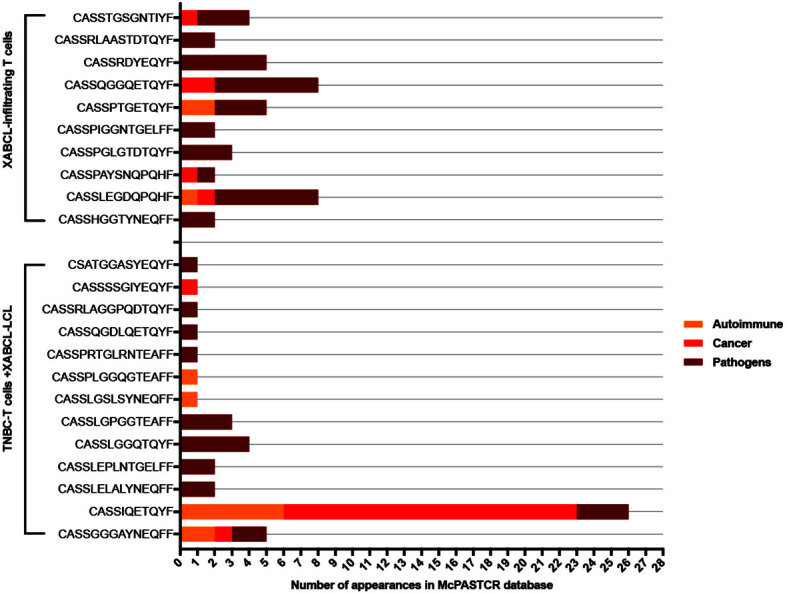
TCR sequences from XABCL-infiltrating T cells and TNBC-T cells expanded in the presence of XABCL-LCL found in the McPAS-TCR database. Ten sequences from the 43 clonotypes (23%) infiltrating the XABCL tissue had been previously reported as TCR sequences recognizing pathogens. From these, 50% had also been described in non-communicable diseases, i.e., cancer and autoimmunity. One sequence had been identified in the three pathologies. From the T cells expanded *in vitro* in the presence of XABCL-LCL, 13 clonotypes were found in the literature: 8, 2, and 1 of these had been exclusively identified in pathogen infections, autoimmunity, and cancer, respectively, and 2 sequences had been described in the three categories, but remarkably the CASSIQETQYF sequence had been identified several times in cancer.

Clonotypes selected both *in vivo* (43 from XABCL tissue) and *in vitro* (73 after XABCL-LCL co-culture) were investigated in the TCR database Mc-PAS-TCR (29) (**Figure 5** and **Supplementary Table 4**). None of the five common clonotypes between XABCL tissue and TNBC-T cells had been previously reported in the literature. Ten of the 43 clonotypes infiltrating the XABCL tissue had been reported; all sequences were described as pathogen-reactive TCR, although some were also related to autoimmunity (two sequences) and cancer (two sequences). One of these sequences was identified in the three categories (CASSLEGDQPQHF). Thirteen of the 73 *in vitro* expanded clonotypes were found in the database: 10 were reported as pathogen-reactive, 2 sequences were exclusively found in autoimmunity, and 1 was found in cancer. Two sequences (CASSGGGAYNEQFF and CASSIQETQYF) were found in the three sets, but it is particularly interesting that the CASSIQETQYF sequence had been reported 6 times in autoimmunity, 3 times in infections, and 17 times in cancer, and in this latter group, it was described as a neoantigen-reactive clonotype.

### 3.4 HLA-Associated Peptides Presented by the XABCL-LCL

The HLA peptidome from XABCL-LCL was analyzed by mass spectrometry. We identified 1,572 HLA-ABC-associated peptides and 1,004 HLA-DR-associated peptides, derived from 1,090 and 444 source proteins, respectively (**Supplementary Table 5**). No EBV-derived peptide was found. After excluding duplicated peptides, i.e., equal sequences with modifications, selecting by length and excluding keratin-derived peptides, 1,392 (HLA-ABC) and 834 peptides (HLA-DR) derived from 1,072 and 353 source proteins remained. Selected peptides fit well to the class I and class II peptide length patterns (**Supplementary Figure 4**). Similarly, the subcellular location of the source proteins given by UniProt (36) corresponded to the different HLA pathways, revealing more source proteins derived from the membrane and the endocytic pathway in the HLA-DR- compared to the HLA-ABC-associated peptides (**Supplementary Figure 4**). Peptides forming nested sets, i.e., peptides sharing a common sequence with varying flanking residues, a classical feature of class II peptides, were observed in the HLA-DR peptidome. A clustering analysis of the peptides was performed by Gibbs (55), revealing different motifs matching the HLA alleles of the XABCL-LCL sample.

We analyzed the XABCL-LCL immunopeptidome, and to exclude commonly presented proteins, the peptide repertoire was compared with LCL peptide repertoires described in the literature from different HLA types. Six studies (39–44) were selected from the literature describing the repertoire of HLA-ABC-derived peptides in LCL (from 7, 11, and 9 different HLA-A, B, and C alleles, respectively). The total number of source proteins used was 26,305. Peptides from the XABCL-LCL were filtered, excluding those derived from proteins that were previously identified in the LCL database generated. The same procedure was performed with the HLA-DR-derived peptidome, and the LCL database generated contained 517 source proteins and 11 different HLA-DR alleles from 10 different studies (45–54). We then analyzed the predicted binding affinities of the filtered peptides (see *Material and Methods*) and selected only those predicted as strong binders. We finally selected 195 peptides predicted as strong binders to HLA-ABC alleles and 223 to HLA-DR alleles. These filtered sequences corresponded to 178 and 102 different proteins, representing 16% and 23% of the total source proteins from the class I- and class II-derived peptides, respectively, obtained from the XABCL-LCL. The KEGG pathway analysis of the source proteins [DAVID (37, 38)] showed that the top 2 results were the antigen processing and presenting (*p* = 1.14E-4) and the phagosome pathways (*p* = 6.6E-3), indicating that the major role of these B cells was antigen presentation.

**Figure 6:**
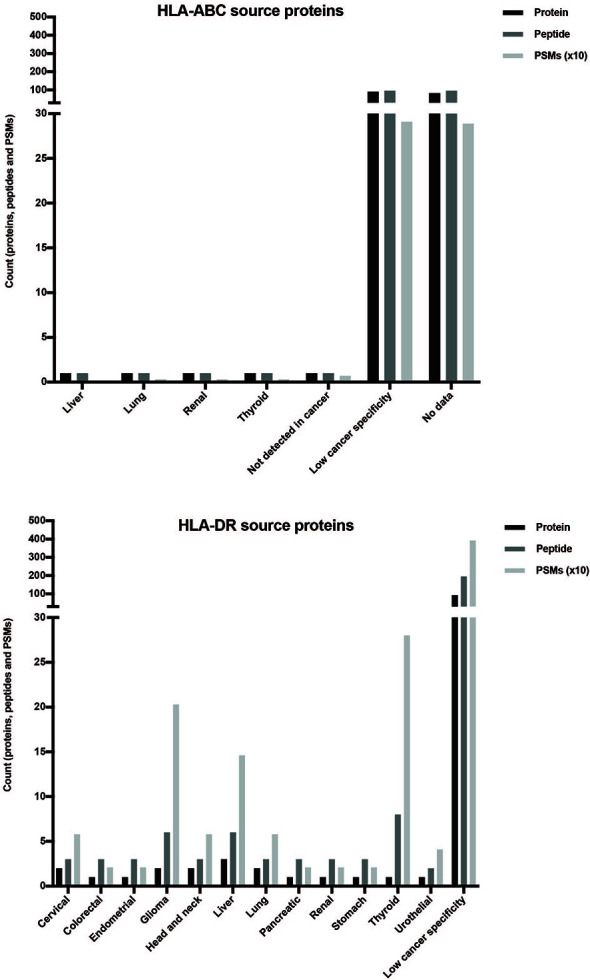
Distribution of the selected proteins obtained from the XABCLLCL HLA repertoire based on their expression in cancer. After comparing the source proteins with those found in the literature, the expression in cancer of 178 (HLA-ABC-associated) and 102 (HLA-DR-associated) proteins was checked by the Human Protein Atlas. Counts of proteins, peptides, and PSM obtained are represented. The highest count in all the measures corresponded to proteins with low cancer specificity, i.e., proteins enriched in many cancer tissues. Two proteins from HLA-ABC-derived peptides showed specificity in certain cancers. Regarding the HLA-DR results, peptides with high PSM values corresponded to specific proteins from thyroid cancer (280 PSM and 8 peptides contained in 1 nested set and derived from 1 protein), glioma (203 PSM and 6 peptides derived from 2 proteins), and liver cancer (146 PSM and 6 peptides derived from 3 proteins).

Finally, the association with cancer of the remaining proteins was checked in the Human Protein Atlas (57–59). For this purpose, the identified proteins and peptides and the number of spectra identified for each peptide (PSM) were used. A higher PSM value is related to a higher abundance of the presented peptide. Most of the analyzed proteins could be found or enriched in many cancer tissues, named as low cancer-specific proteins (**Figure 6** and **Supplementary Table 6**). In the HLA-ABC repertoire, there were only two cancer-enriched proteins and a high number of proteins without enough available data in the database. Interestingly, several proteins specific for some cancers such as thyroid cancer (1 protein, 8 peptides, and 280 PSM), glioma (2 proteins, 6 peptides, and 203 PSM), and liver cancer (3 proteins, 6 peptides, and 146 PSM) were identified in the HLA-DR peptide repertoire.

## 4 Discussion

Tumors impair the immune system action generating an adverse microenvironment by expressing immune checkpoints that overall favor a regulatory immune response (60). ICPI treatments such as pembrolizumab or nivolumab and ipilimumab against PD-1 and CTLA-4 receptors, respectively, have increased the survival of cancer patients (61). At the same time, blocking the immune checkpoint molecules can trigger the disruption of immune homeostasis and, consequently, lead to immune-related adverse events [known as irAEs (62)]. The infection by some human viruses itself has also been proposed to be related with autoimmune and inflammatory processes in several cases (24, 25, 63). Thus, the presence of EBV+ B cells, as well as other virus-infected cells, in an immunomodulated microenvironment together with ICPI treatment may be an additional factor with a role in the development of irAEs.

In the last years, different groups have performed analysis of the association between infection by different species of human herpesvirus (HHV) such as EBV (or HHV-4) (64) and cytomegalovirus (CMV or HHV-5) (65), or retrovirus such as human T-lymphotropic virus (HTLV) (66), and the tumor outcomes. However, these studies are all focused on the direct role of the viruses in the development of tumors but not in the contribution of the antivirus responses to the antitumor response. In this research, we have been able to study different immune-related factors associated with the presence of EBV+ B cells among TIL. We have studied a TNBC biopsy obtained before neoadjuvant chemotherapy and a XABCL lymphocytic tumor generated from a simultaneously obtained biopsy, as well as T cells derived from the TNBC (TNBC-T cells) and the EBV+ B cell line derived from the XABCL (XABCL-LCL).

IHC of the biopsy of the patient first revealed that most of the CD3+ T cells found in the histological section were CD4+ T cells colocalized with CD20+ cells, while CD8+ T cells were localized nearest to the tumor in the infiltrating front of neoplasia. This would suggest a possible *in situ* interaction between B cells and Th. Since B cells can act as professional APC, the presence of B cells, even if they are not tumor-specific, can contribute to tune the tumor landscape. The role of B cells as a putative inductor of the T-cell response in the tumoral microenvironment in BC is still unclear (67).

The XABCL generated from the TNBC biopsy resulted in a lymphocytic tumor; XABCL-LCL was CD19+, CD20+, CD21−, and CD38+, an activated germinal center B-cell phenotype. We confirmed that the XABCL-LCL, as well as the PBMCs of the patient, was EBV+. This is not surprising since up to 40% of Hodgkin’s lymphomas are EBV-associated and LCLs can be generated *in vitro* using EBV. Considering that EBV is detected in more than 90% of the adult population of the world (68) and that it has the ability to be maintained for life in a latent stage, it is not unexpected that among TIL there were EBV+ B cells. The development of EBV+ lymphocytic neoplasms in PDX derived from breast cancer had also been previously described (10). This can be explained by the lack of a mature immune system in the PDX models, which are NSG mice, and the evidence that EBV infection more frequently leads to lymphoproliferative disorders in immunosuppressive contexts (20, 69, 70). We are not aware that these XABCLs have been used before as a model to investigate the role of tumor-infiltrating EBV+ B cells.

The BCR analysis of the XABCL revealed a unique BCR sequence representing the major fraction in both the tissue and the LCL. This confirmed that the conversion into a lymphocytic tumor was produced in the mice and not by the establishment of an LCL *in vitro*. We analyzed the BCR repertoire in the initial patient biopsy, and this BCR sequence was found in the TNBC, among many other BCR sequences.

Interestingly, the histological section obtained from the first pass of the XABCL tumor revealed some CD3+ T cells infiltrating the tumor. TCR sequencing confirmed that some T cells were still maintained after the third XABCL pass. Forty-three clonotypes were obtained and 18 of these were also found in the TNBC frozen tissue or in the expanded TNBC-T cells. The maintenance of T cells in the XABCL during the three passes in mice may be the result of stimulation by the XABCL B cells, which expressed the HLA class I and class II molecules as well as the costimulatory molecules CD80, CD86, and PD-L1. An increased expression of the HLA molecules is usually observed in EBV+ B cells (63). Moreover, a different pattern of TCR was observed in the TNBC samples compared with the XABCL, suggesting that certain clonotypes were being selected by XABCL B cells. Therefore, we used TNBC-T cells for co-culture with irradiated XABCL-LCL to study if a T-cell selection could also be observed *in vitro.* Upon interaction with the XABCL-LCL, 73 clonotypes were identified and a decrease in TCR diversity was observed. Two of the sequences expanded were previously found in the XABCL, i.e., CARSTNGELFF (TRBV28) and CSARVPGLPDTQYF (TRBV20-1). Certain clonotypes found in the initial cultures were maintained in the XABCL-LBL-expanded TNBC-T cells, but not in the control. Thus, these results altogether suggest that some EBV+ B cells may be able to stimulate T cells from TIL, inducing a certain selection of the TCR repertoire.

The peptide repertoire presented by HLA-ABC and HLA-DR in the XABCL-LCL was analyzed. We did not find EBV-derived peptides neither in the class I nor in the class II analysis. This is not very surprising since EBNA1 is the only viral protein expressed in all EBV-associated malignant diseases (71). The subcellular location of source proteins showed a pattern common to B-cell line repertoires (53, 54). The clustering analysis obtained revealed two binding motifs matching the HLA haplotype of the patient. It is worth mentioning that in the HLA-DR study, the most abundant source protein was GAPDH, and although it is a commonly found protein, it is not that frequent to find HLA-DR-associated GAPDH peptides in high abundance. A relationship between GAPDH with breast cancer has been described (72, 73); therefore, it could be interesting to further analyze the relevance of the GAPDH-derived peptides presented by these cells.

There are several studies of the HLA class I and class II repertoire using LCL, but these are transformed *in vitro* while the XABCL B cells used in this study were infected and transformed by the EBV *in vivo*. Therefore, there could be differences in the peptide repertoire between these cells and the typical LCL. In order to study this, we excluded the source proteins from the peptides obtained from LCL found in the literature, independently for the class I (39–44) and the class II alleles (45–54). The resulting protein pool represented 16% and 23% of the total source proteins obtained from the HLA-ABC and the HLA-DR peptidomes, respectively. These proteins were mostly involved in the antigen processing and presentation and in the phagosome pathways. This indicates that these cells may present some different proteins compared with other LCL and that these B cells still can act as APC.

Many of the selected sequences were derived from proteins that can be present in many cancer tissues, catalogued as proteins with low cancer specificity. Nevertheless, some proteins enriched in cancer were also found, especially in the HLA-DR selected peptides. Thus, in a tumoral microenvironment, peptides derived from autologous proteins as cancer-enriched proteins can be presented by these B cells. In the future, these peptides should be used separately *in vitro* to analyze their capacity to stimulate cancer-infiltrating T cells.

Several features of EBV+ B cells make them relevant in the tumor context. Because tumor cells are known to downregulate the HLA molecules to escape from immune response, the activation and maintenance of both CD4+ and CD8+ T cells in the tumor site is usually the responsibility of APC. The role of B cells as modulators of the antitumoral response has been latterly considered (74). However, EBV+ B cells in a latent stage are memory-like B cells activated by signaling pathways similar with those produced by the CD40L-mediated activation (75), in the absence of T-cell signals and regardless of their BCR specificity. Moreover, EBV+ B cells in a latent stage have an abnormal transcriptome (76). On the other hand, although EBV-specific T cells are maintained throughout life, participating in a persistent control of the infection, the immunomodulatory capacities of the tumor cells affecting the microenvironment may give the EBV a chance to start an infectious stage and to escape the immune response. Two recent studies have reported fatal inflammations [encephalitis (25) and myocarditis (24)] in post-ICPI melanoma patients, in which EBV-specific T cells were involved. Even so, the antigens responsible for these dysregulations may not be limited to EBV-derived antigens.

In summary, this is a first proof of concept that gives relevance to EBV+ B cells present in the tumor site. We have not only demonstrated their presence but also that they are in an activated status and able to stimulate T cells from TIL. Our data also evidence that EBV+ B cells can modulate the TCR repertoire as a certain pattern has been identified and some of the TCR sequences maintained had been already reported in the literature. Certain uncommon peptides presented by HLA-ABC and HLA-DR molecules have been also identified. Thus, to take profit of the XABCL tumors can be a tool to understand the role of EBV+ B cells in the tumor infiltrates and how they can contribute to different patient outcomes by shaping the TCR repertoire.

## Data Availability Statement

The datasets presented in this study can be found in online repositories. TCR sequencing data from the TNBC-derived T cell cultures are available as SRA accessions in the BioProject PRJNA75917. The TCR and BCR HTS from TNBC and XABCL biopsies are available as an ImmuneAccess Project (DOI https://doi.org/10.21417/AA2021FI). The mass spectrometry proteomics data have been deposited to the ProteomeXchange Consortium via the PRIDE (77) partner repository with the dataset identifier PXD028646.

## Author Contributions

Conceptualization: AA and MM. Methodology: AA, MM, CR-M, MC, EM, and YA. Investigation: AA, VP, RR, EM, and YA. Resources: VP, RR, CB, EZ, JA, and MC. Writing—original draft: AA and MM. Writing—review and editing: AA, MC, JC, and MM. Supervision: JC and MM. All authors contributed to the article and approved the submitted version.

## Funding

This project was funded by Roche Farma, S.A. grant SP181123001 and the Spanish Ministry of Science, Innovation and Universities grant RTI2018-097414-B-I00. Partial financial support was received from the “El Paseíco de la Mama” 2015. This study received partial funding from Roche Farma, S.A. The funders were not involved in the study design, collection, analysis, interpretation of data, the writing of this article, or the decision to submit it for publication.

## Conflict of Interest

The authors declare that the research was conducted in the absence of any commercial or financial relationships that could be construed as a potential conflict of interest.

## Publisher’s Note

All claims expressed in this article are solely those of the authors and do not necessarily represent those of their affiliated organizations, or those of the publisher, the editors and the reviewers. Any product that may be evaluated in this article, or claim that may be made by its manufacturer, is not guaranteed or endorsed by the publisher.
