# Supplementary material for: Epstein–Barr Virus+ B Cells in Breast Cancer Immune Response: A Case Report
Source: Front Immunol. 2021 Nov 16;12:761798. doi: 10.3389/fimmu.2021.761798 (PMC8637110; doi:10.3389/fimmu.2021.761798)
Supplement: Supplementary file 1 [file DataSheet_1.docx]

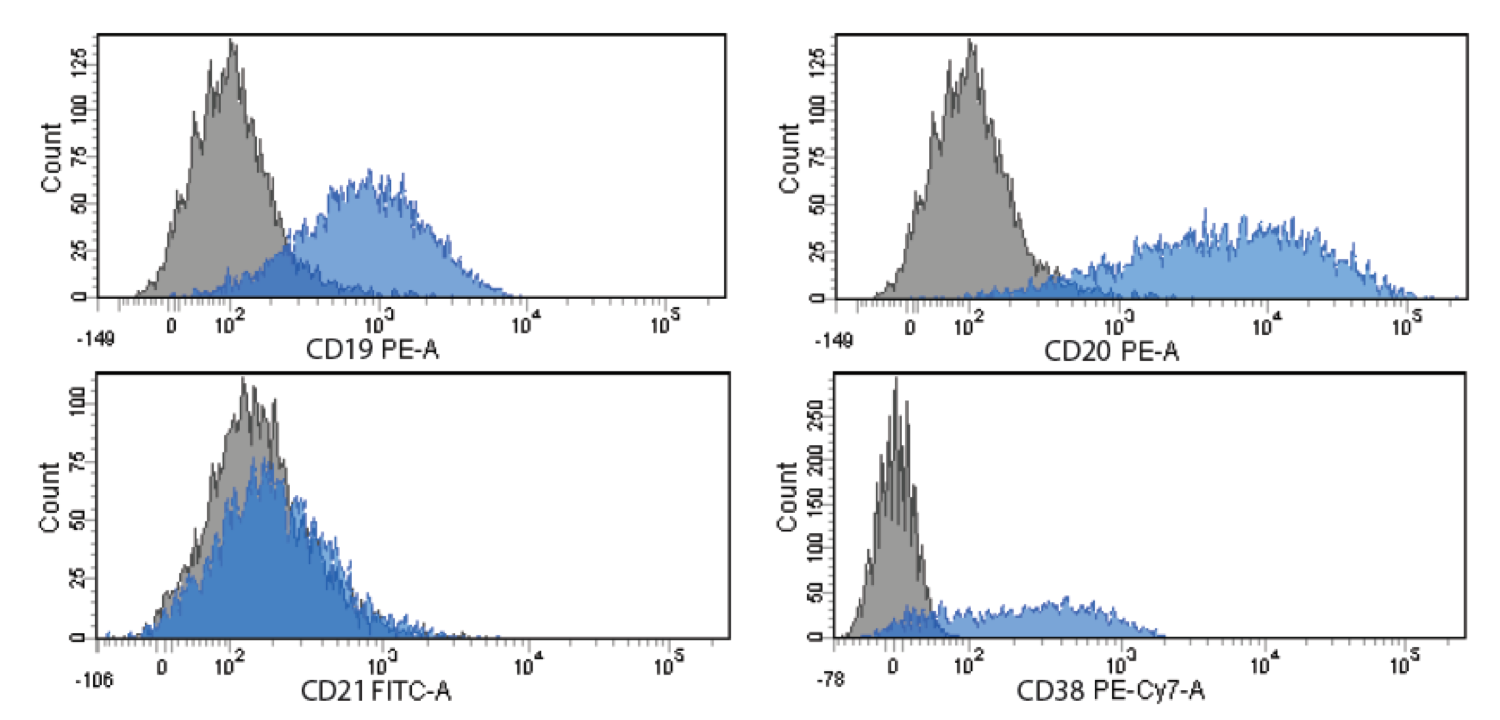

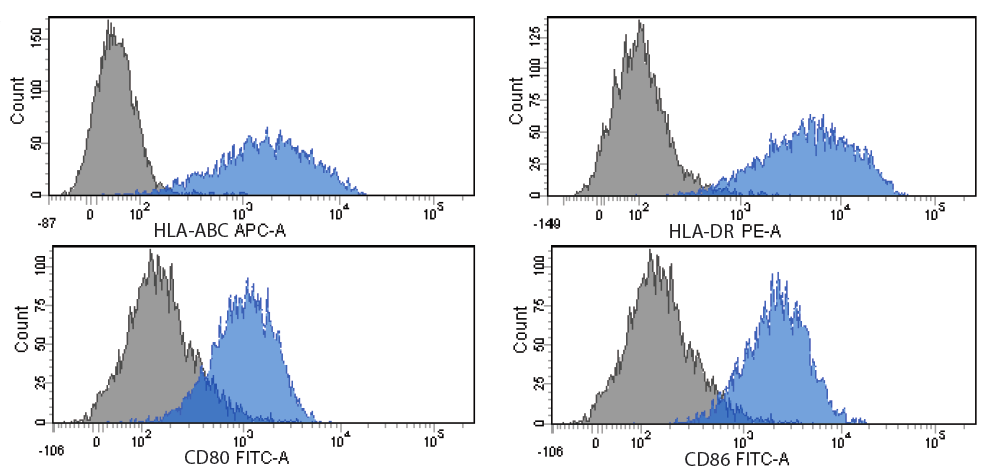

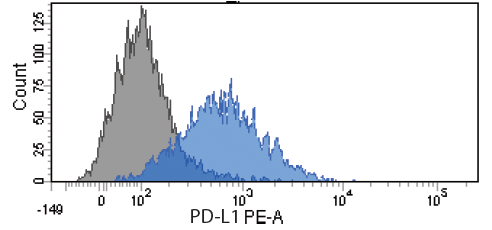


**Supplementary Figure 1. The XABCL-LCL B cells showed a memory-like phenotype and expressed antigen-presenting and costimulatory molecules.** Anti-human CD19-PE, CD20-PE, CD21-FITC and CD38-PE-Cy7, HLA-ABC-APC, HLA-DR-PE, CD80-FITC, CD86-FITC and PD-L1-PE antibodies were used. XABCL-LCL expressed CD19, CD20 and CD38 but were negative for the CD21 receptor. HLA-ABC and HLA-DR, as well as the costimulatory molecules CD80, CD86 and PD-L1 were also expressed, confirming that these cells still had antigen-presenting capacities.

**CD20**

**CD3**

B

C

A


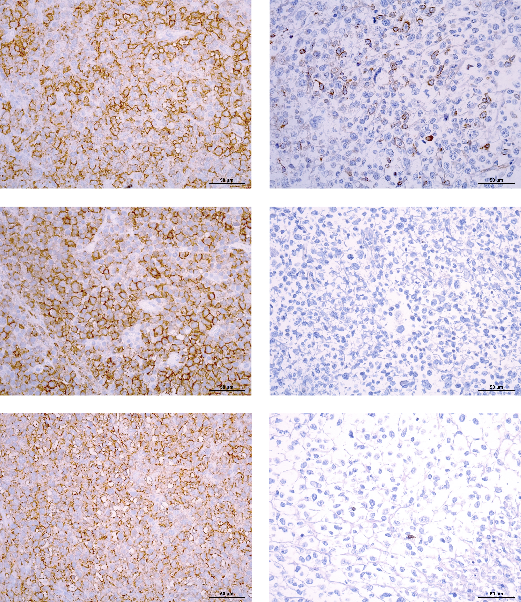


**Supplementary Figure 2. Infiltration of CD3+ T cells was observed in the XABCL biopsy obtained from the first mouse.** IHC using anti-human CD20 and anti-human CD3 was performed in three slices from biopsies corresponding to different passes into mice. (A) was obtained from the first mouse inoculated with the patient biopsy, (B) from the second pass and (C) from the last pass. Most of the cells were already CD20+ at the first pass and an infiltration of several CD3+ T cells was observed. In (B) and (C) CD3+ T cells could not be observed by IHC. Tissue block from the third pass (C) was used to perform IGHV and TRBV sequencing.

A

**TNBC-T cells + OKT3**


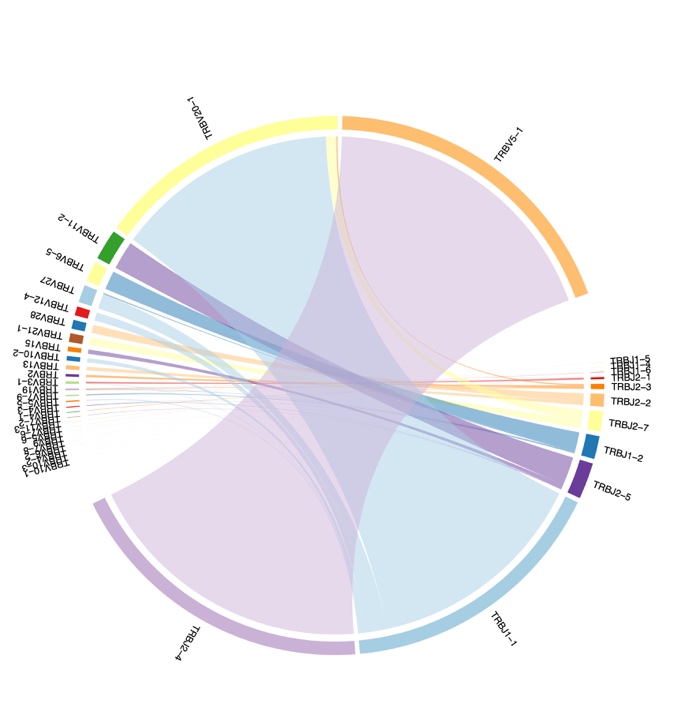


**TNBC- T cells Initial Culture**


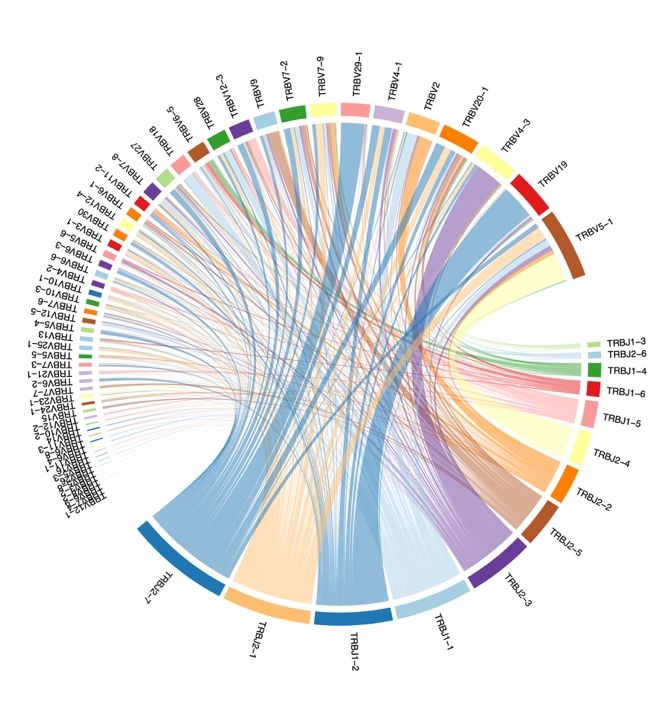


**TNBC-T cells + XABCL-LCL**


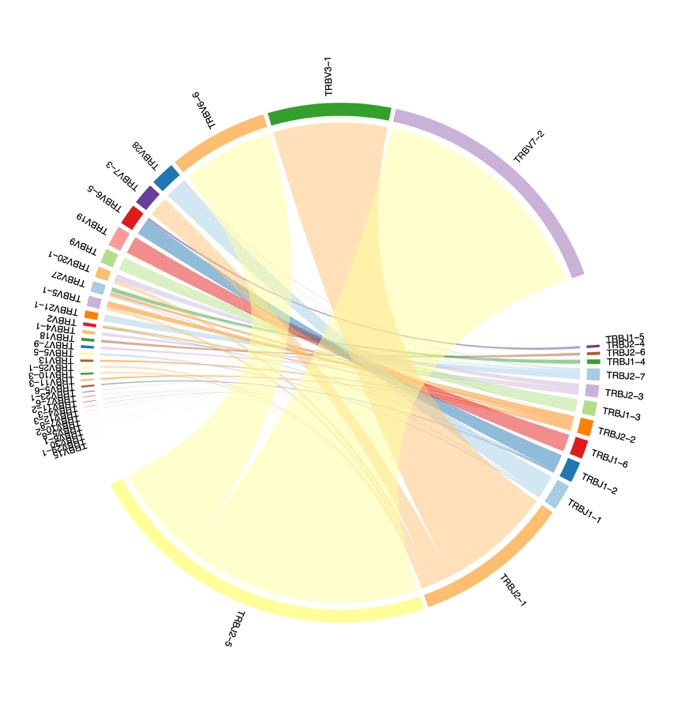


**Supplementary Figure 3. TCR repertoire and T cell phenotype changes after TNBC-T cell coculture with XABCL-LCL.** (A) V-J usage of samples calculated by counting the read frequency (weighted). The initial TNBC-T cell culture revealed a higher number of V-J recombinations. Cells expanded with OKT3 (anti-CD3) showed a different pattern than cells after co-cultures with XABCL-LCL. (B) The normalized fractions of the two CDR3 sequences that were also found infiltrating the XABCL-562. The CARSTNGELFF CDR3 sequence represented a smaller fraction in the control (0.0012) compared with that in the initial cultures (0.0088), but a higher fraction after co-cultures (0.027). The CSARVPGLPDTQYF CDR3 sequence was over-expanded in the control (0.0217) in comparison with the initial culture (0.0094) and with the co-cultures (0.0042), representing a smaller fraction in this latest. The number of reads of both sequences in each set is shown above the bars. (C) Phenotype of TNBC-T cells in the initial culture (10 days) and after XABCL-LCL co-culture (irradiated at 60Gy). The most abundant subset in the first sample was DN (CD4-CD8- T cells) in a 41.8%, followed by the CD8+ T cells in a 40.6%. In the T cells co-cultured, CD8+ T cells were present in a 52.1% and CD4+ T cells in a 43.6%, while the DN subset decreased to a 1.34%.

B

C


**Supplementary Figure 4. Peptide length distribution and localization of peptides eluted from the XABCL-LCL HLA-ABC and HLA-DR molecules.** (A) Peptide length distribution of the 1392 and 834 peptides obtained from the HLA-ABC and HLA-DR molecules, respectively, after pre-filtering. The class I peptides presented a high dominance of 9-mer peptides, and the class II peptides presented a Gaussian distribution with a highest frequency of 15-mer peptides, denoting reliable repertoires. (B) Intracellular distribution of the source proteins. Values are the percentage of proteins for each intracellular localization, as described in the Uniprot database. c, cytosol; M, membrane; Lys/End, lysosome or endosome; E/EM, exogenous or extracellular matrix; g, Golgi; ER, endoplasmic reticulum; N, nuclear; mit, mitochondria; and ND; not defined.

A

B

**HLA-ABC derived peptides**

**HLA-DR derived peptides**
